# Predictors of ventilatory outcome in cervical spinal injuries

**DOI:** 10.1186/cc13640

**Published:** 2014-03-17

**Authors:** HT Wang, DW Williamson, MA Albert

**Affiliations:** 1 HopitalSacre-Coeurde Montreal, Canada

## Introduction

Spinal cord injuries affect 50 persons per million every year in North America [1], with over 50% occurring at the cervical level [2]. Cervical spinal cord injuries (CSI) are at particular risk for mechanical ventilation (MV), pulmonary complications and increased length of hospital stay. A few small cohort studies looked at predictors of MV [3-6], and to our knowledge there are no studies addressing factors associated with prolonged MV. The purpose of this study was to compare known clinical predictors of MV and determine predictors of prolonged MV.

## Methods

We conducted a retrospective chart review of consecutive CSI admitted between 1 January 2005 and 1 March 2009. We recorded data related to the injury, the duration of MV, respiratory complications, ICU and hospital length of stay and patients' outcomes. A review of the literature identified known predictors (ASIA level, ISS, level of injury, and so forth). Univariate and multivariate logistic regression were used to identify predictors of MV and prolonged MV.

## Results

Of the 208 patients, 82% were male and the mean age was 51 years. Hospital mortality was 8.7%. Main causes of injury were motor vehicle accidents (39.7%) and falls (43.2%). Injuries below C4 level represented 51.5% of the population. A complete loss of motor function (ASIA level A and B) was found in 34.9% of patients. The mean and median ISS score was 20.7. In total, 78 patients required MV (37.5%) and 30 patients required prolonged MV (14.4%). After multivariate analysis, four predictors of MV were identified: pneumonia (OR = 52.83); ISS score >22 (OR = 4.09); age (OR = 1.02); level C1 to C4 (2.34); and two predictors of prolonged MV: ASIA score A and B (OR = 5.57) and pneumonia (OR = 8.76).

## Conclusion

In our study ISS, cervical level and age were associated with MV but not with the need for prolonged MV, whereas pneumonia was an independent risk factor for both. This is a potentially preventable risk factor where specific strategies can be applied to improve patients' outcome.
